# BTBD10 is a Prognostic Biomarker Correlated With Immune Infiltration in Hepatocellular Carcinoma

**DOI:** 10.3389/fmolb.2021.762541

**Published:** 2022-01-04

**Authors:** Jianhui Li, Xiaojuan Tian, Ye Nie, Ying He, Wenlong Wu, Xinjun Lei, Tianchen Zhang, Yanfang Wang, Zhenzhen Mao, Hong Zhang, Xuan Zhang, Wenjie Song

**Affiliations:** ^1^ Xi’an Medical University, Xi’an, China; ^2^ Department of Hepatobiliary Surgery, Xijing Hospital, Fourth Military Medical University, Xi’an, China; ^3^ Operating Room, Xijing Hospital, Fourth Military Medical University, Xi’an, China

**Keywords:** BTBD10, hepatocellular carcinoma, prognosis, biomarker, immune infiltration

## Abstract

**Background:** BTBD10 serves as an activator of Akt family members through decreasing the protein phosphatase 2A-mediated dephosphorylation. The present study attempted to investigate the prognostic value of BTBD10 in hepatocellular carcinoma (HCC), specially, its relationship with tumor-infiltrating lymphocytes (TILs).

**Methods:** BTBD10 expression was evaluated in HCC using The Cancer Genome Atlas (TCGA) and Xijing Hospital database, and verified in HCC cell lines. Cox analyses were performed to analyze independent prognostic risk factors for HCC. The optimal cut-off value of BTBD10 was calculated, by which all patients were divided into two groups to compare the overall survival (OS). The signaling pathways were predicted, by which BTBD10 may affect the progression of HCC. To investigate the impact of BTBD10 on HCC immunotherapy, correlations between BTBD10 and TILs, immune checkpoints, m6A methylation-related genes and ferroptosis-related genes were assessed. The distribution of half-maximal inhibitory concentration (IC50) of diverse targeted drugs was observed based on the differential expression of BTBD10.

**Results:** BTBD10 expression was higher in HCC tissues and cell lines than that of normal liver tissues and cells. The patients with high expression of BTBD10 showed a worse OS, as compared to that of BTBD10 low-expressing group. Cox analyses indicated that BTBD10 was an independent prognostic risk factor for HCC. Several molecular pathways of immune responses were activated in HCC patients with high-expressing of BTBD10. Furthermore, BTBD10 expression was demonstrated to be positively correlated with tumor-infiltrating B cells, T cells, macrophages, neutrophils and dendritic cells. Meanwhile, the expression of BTBD10 was synchronized with that of several m6A methylation-related genes, ferroptosis-related genes and immune checkpoints. The IC50 scores of Sorafenib, Navitoclax, Veliparib, Luminespib, and Imatinib were found to be lower in BTBD10 high-expressing HCC group.

**Conclusion:** BTBD10 negatively regulates tumor immunity in HCC and exhibits adverse effect on the prognosis of HCC, which could be a potential target for immunotherapy.

## Introduction

The high mortality and recurrence of hepatocellular carcinoma (HCC) made it the second leading cause of cancer-related death in the world (Chen et al., 2016; Sung et al., 2021). Although current treatments for HCC, including interventional or radiofrequency ablation (Pérez-Romasanta et al., 2021), chemotherapy or targeted therapy (Cheon et al., 2021), surgical resection (Gunasekaran et al., 2021), and liver transplantation (Hughes and Humar, 2021), etc., exhibit respective therapeutic effect, the overall survival (OS) of HCC is still unsatisfied (D'Avola et al., 2021). Therefore, there is an urgent need to explore new effective biomarkers and/or potential molecular targets for anti-tumor therapy to improve the treatment strategy of HCC patients with different pathological stages.

BTB/POZ domain-containing protein 10 (BTBD10) is located on human chromosome 11p15.2 and consists of nine exons spanning a 1,428 bp open reading frame (ORF) that encodes a 475-amino acid protein (Chen et al., 2004). BTBD10 plays a major role as an activator of AKT family members by inhibiting PPP2A-mediated dephosphorylation, thereby keeping AKTs activated (Nawa et al., 2008). For example, BTBD10 overexpression inhibited protein phosphatase 2A-mediated Akt dephosphorylation and G93A-superoxide dismutase 1-induced motor neuron death (Nawa et al., 2012). BTBD10 regulated pancreatic beta cell proliferation and apoptosis *via* activation of Akt signaling pathway (Wang et al., 2011). Specially, BTBD10 has been found to be associated with tumor progression. The BTBD10 subunit (KCTD20) enhanced the proliferation and invasion of non-small cell lung (NSCLC) cancer by increasing the phosphorylation level of Akt (Zhang et al., 2017a). However, the possible biological function of BTBD10 in HCC and its prognostic value have not been investigated yet.

In this study, we explored the expression of BTBD10 in HCC and analyzed the relationship of BTBD10 expression with the prognosis of HCC patients. Furthermore, we unraveled the potential molecular mechanisms, by which BTBD10 exhibited an obvious impact on the targeting and/or immunotherapy of HCC.

## Patients and Methods

### HCC Sample Collection and Follow-Up Study

Sixty patients with HCC who underwent surgical excision at Xijing Hospital in 2017 were randomly selected for this study. Three patients with missing data were excluded, leaving 57 patients for analysis. None of the patients had received preoperative radiotherapy or chemotherapy. The pathological results of all patients were HCC. Data from Xijing Hospital included 57 tumor samples and 20 normal tissue samples. This study was approved by the Xijing Hospital Ethics Committee (KY20172013-1) in accordance with the criteria of the Helsinki Declaration.

### RNA Extraction and Gene Expression Measurement

RNA was extracted from all tissue samples, normal human liver cell lines (MIHA) and the human hepatocellular carcinoma cell lines (HepG2, Hep3B, HUH7, LM3, MHCC97H, SNU-368 and SNU-739), according to the instructions of the kit. Real-time quantitative fluorescence PCR (qRT-PCR) assay to detect the expression levels of BTBD10, PD-1, PD-L1, PD-L2, CTLA4 and GAPDH. Using GAPDH as an internal control, the 2^−ΔΔCt^ method was used to calculate the relative expression levels of each gene in the samples. All cell lines were purchased from the National Collection of Authenticated Cell Cultures (Shanghai, China). Primer sequences used for amplification are shown in Table 1.

**TABLE 1:** Primer sequences of quantitative real-time polymerase chain reaction (qRT-PCR).

Genes		Primer sequences (5′-3′)
BTBD10	F	GGA​CGG​CCT​CAT​CCC​TAT​GAT
	R	CTT​TAG​CAA​TAC​GCG​AGG​AAG​TA
PD-L1	F	TGG​CAT​TTG​CTG​AAC​GCA​TTT
	R	TGC​AGC​CAG​GTC​TAA​TTG​TTT​T
PD-1	F	CCAGCCCCTGAAGGAGGA
	R	GCC​CAT​TCC​GCT​AGG​AAA​GA
PD-L2	F	ACC​CTG​GAA​TGC​AAC​TTT​GAC
	R	AAG​TGG​CTC​TTT​CAC​GGT​GTG
CTLA4	F	GCC​CTG​CAC​TCT​CCT​GTT​TTT
	R	GGT​TGC​CGC​ACA​GAC​TTC​A
GAPDH	F	AGA​AGG​CTG​GGG​CTC​ATT​TG
	R	AGG​GGC​CAT​CCA​CAG​TCT​C

### Downloading and Screening Data

UALCAN (http://ualcan.path.uab.edu/index.html) was used to predict the expression difference of BTBD10, the relationship between BTBD10 and tumor stage, grade, TP53 mutation and DNA methylation. The sequencing data and corresponding clinical data of HCC patients were downloaded from TCGA database. Patients with a survival time less than 30 days and those missing clinicopathological parameters were excluded, leaving 343 tumor patients and 50 normal patients for analysis.

### Survival Analysis

The optimal cut-off value of BTBD10 was calculated according to the Youden index [(sensitivity + specificity) −1], and the HCC samples were divided into two groups: high expression and low expression of BTBD10. Kaplan-Meier (K-M) method was used to compare the survival differences between the two groups and determined by log-rank test. *p* < 0.05 was considered significant.

### Enrichment Analysis

In order to understand the carcinogenic mechanism of BTBD10, we used GSEA software (4.1.0 version) to perform Gene Ontology (GO) and Kyoto Encyclopedia of Genes and Genomes (KEGG) analysis to explore the role of BTBD10 in the progression of HCC (*p* < 0.05).

### Immunoassay

To determine the association between BTBD10 and TILs, we used the Tumor IMmune Estimation Resource (TIMER, https://cistrome.shinyapps.io/timer/) and TCGA data to analyze the relationship between BTBD10 expression and TILs. The relationship between BTBD10 and immune checkpoints in different groups was evaluated to further analyze the effect of BTBD10 on TILs. Potential ICIs response was predicted with Tumor Immune Dysfunction and Exclusion (TIDE) algorithm. The Wilcoxon test was used to explore the relationship between BTBD10 expression with m6A methylation-related genes and ferroptosis-related genes to guide clinical immunotherapy.

### IC50 Scores

The half-maximal inhibitory concentration (IC50) is an important indicator for evaluating the efficacy of a drug or the response of a sample to treatment. Using the largest publicly available pharmacogenomics database, Genomics of Drug Sensitivity in Cancer (GDSC), the sample-based transcriptome predicts the response of each sample to the targeting and/or immunotherapy of HCC.

### Statistical Analysis

Unpaired t test was performed for the expression differences of BTBD10 using GraphPad Prism 6 software. Cox analysis was used to determine the independent prognostic risk factors of HCC patients. Survival differences were expressed by K-M curve and determined by log-rank test. Spearman correlation analysis was used for correlation analysis. Related R packages included “forestplot”, “rms”, “pheatmap”, “ timeROC ”, “GSEABase ”, “ggstatsplot” and “ggplot2”. A p-value of less than 0.05 was considered statistically significant.

## Results

### Identification of BTBD10 Expression in HCC Tissues and Cell Lines

**FIGURE 1:**
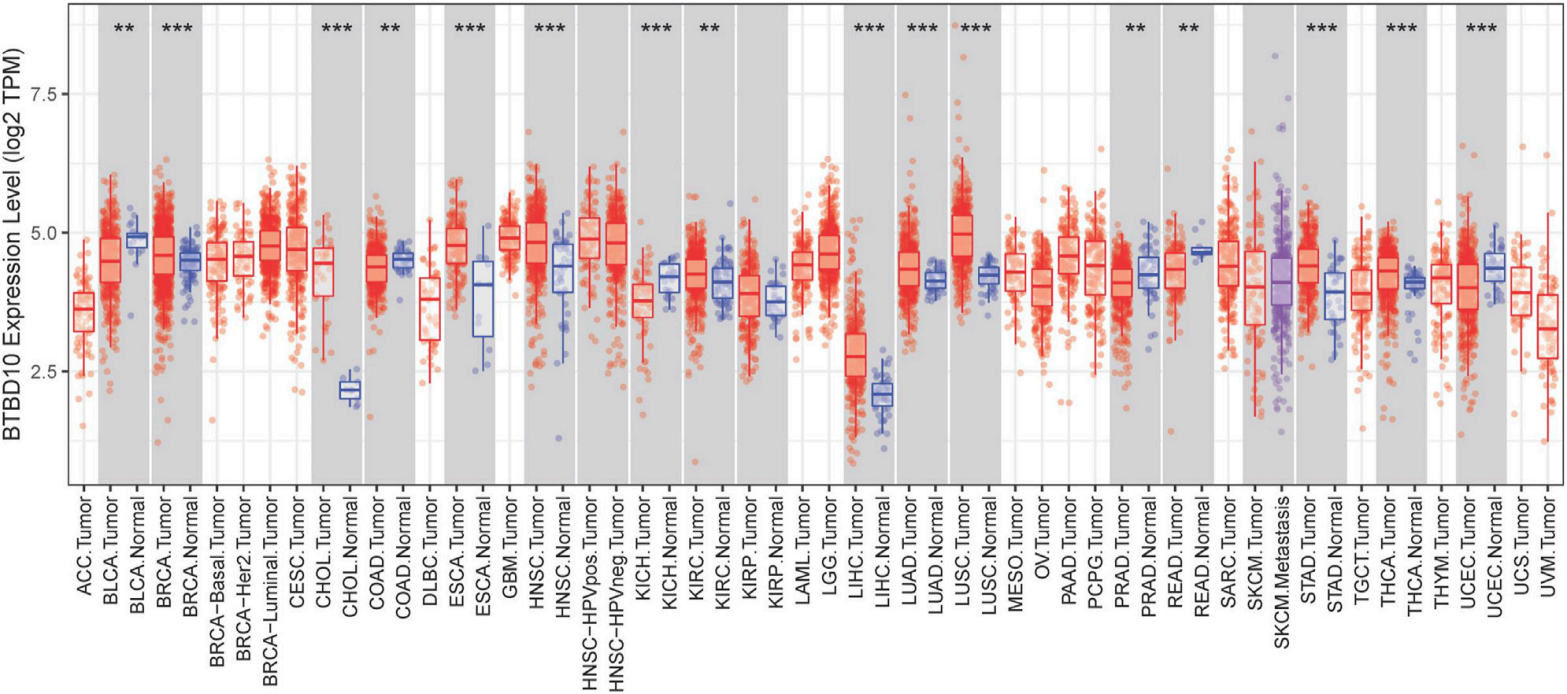
BTBD10 expression levels in various cancer tissues determined using the TIMER analysis. *p* < 0.001 = ***, *p* < 0.01 = **, and *p* < 0.05 = *.

**FIGURE 2:**
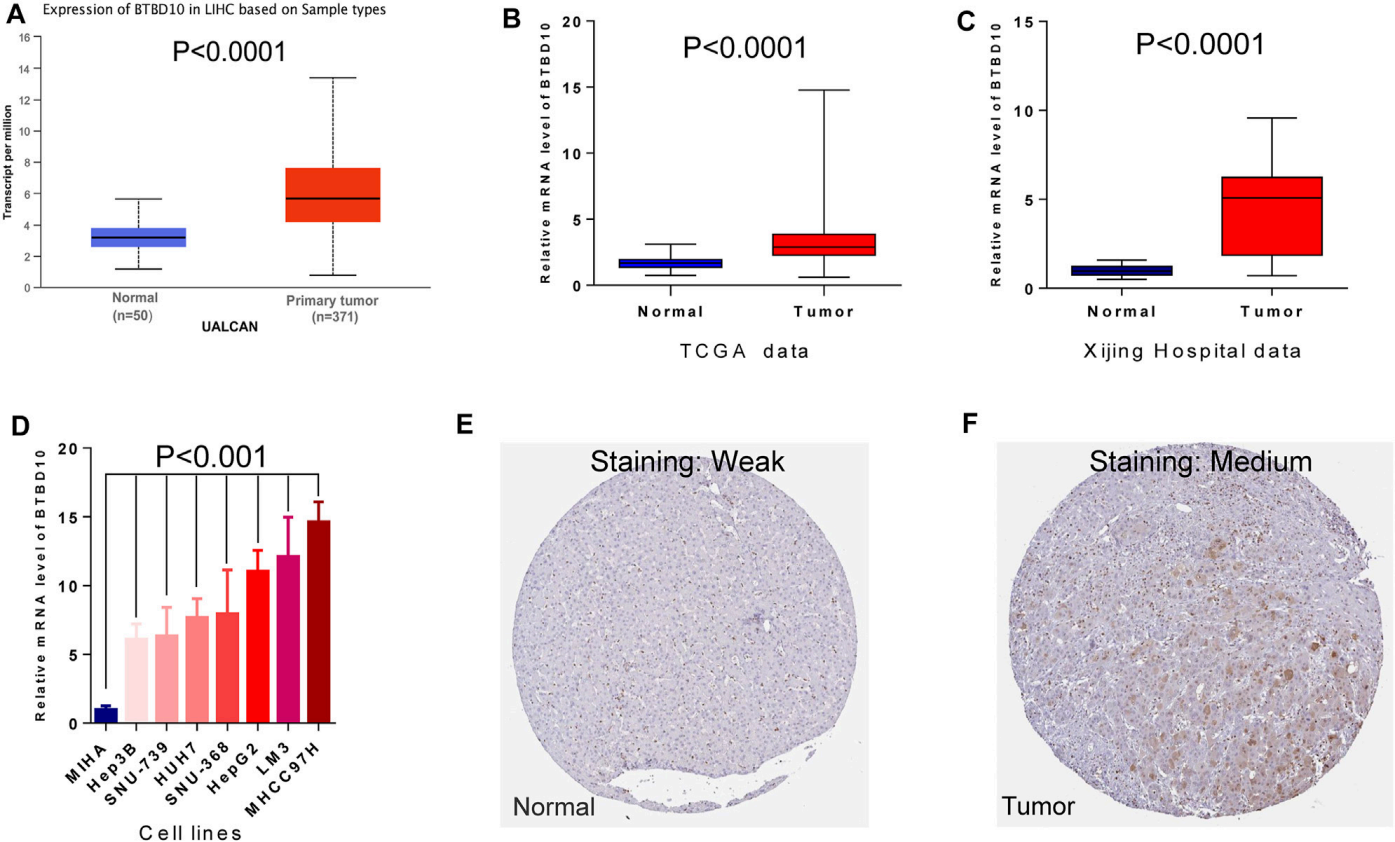
Differences in expression of BTBD10: **(A)** UALCAN data; **(B)** TCGA data; **(C)** Xijing Hospital data; **(D)** cell lines. Representative images of immunohistochemical staining for BTBD10 in normal liver tissues and HCC tissues from the Human Protein Atlas: **(E)** normal tissues; **(F)** tumor tissues.

According to the prediction of TIMER data, the expression of BTBD10 mRNA in 11 types of tumor tissues (including HCC tissues) was significantly higher than that in normal tissues (Figure 1). Meanwhile, we confirmed the higher expression of BTBD10 mRNA in HCC tissues with the data from UALCAN and TCGA database (Figures 2A,B). Furthermore, qRT-PCR was performed to verify the expression of BTBD10 mRNA in HCC tissue and cell lines. The result demonstrated the higher expression of BTBD10 mRNA in HCC tissues obtained from Xijing Hospital (Figure 2C) and HCC cell lines (HepG2, Hep3B, HUH7, LM3, MHCC97H, SNU-368 and SNU-739) (Figure 2D). Immunohistochemical results of Human Protein Atlas (HPA) also indicated the higher expression of BTBD10 protein in HCC tissues than that in normal liver tissues (Figures 2E,F).

### BTBD10 Expression Affects the Prognosis of HCC Patients

**FIGURE 3:**
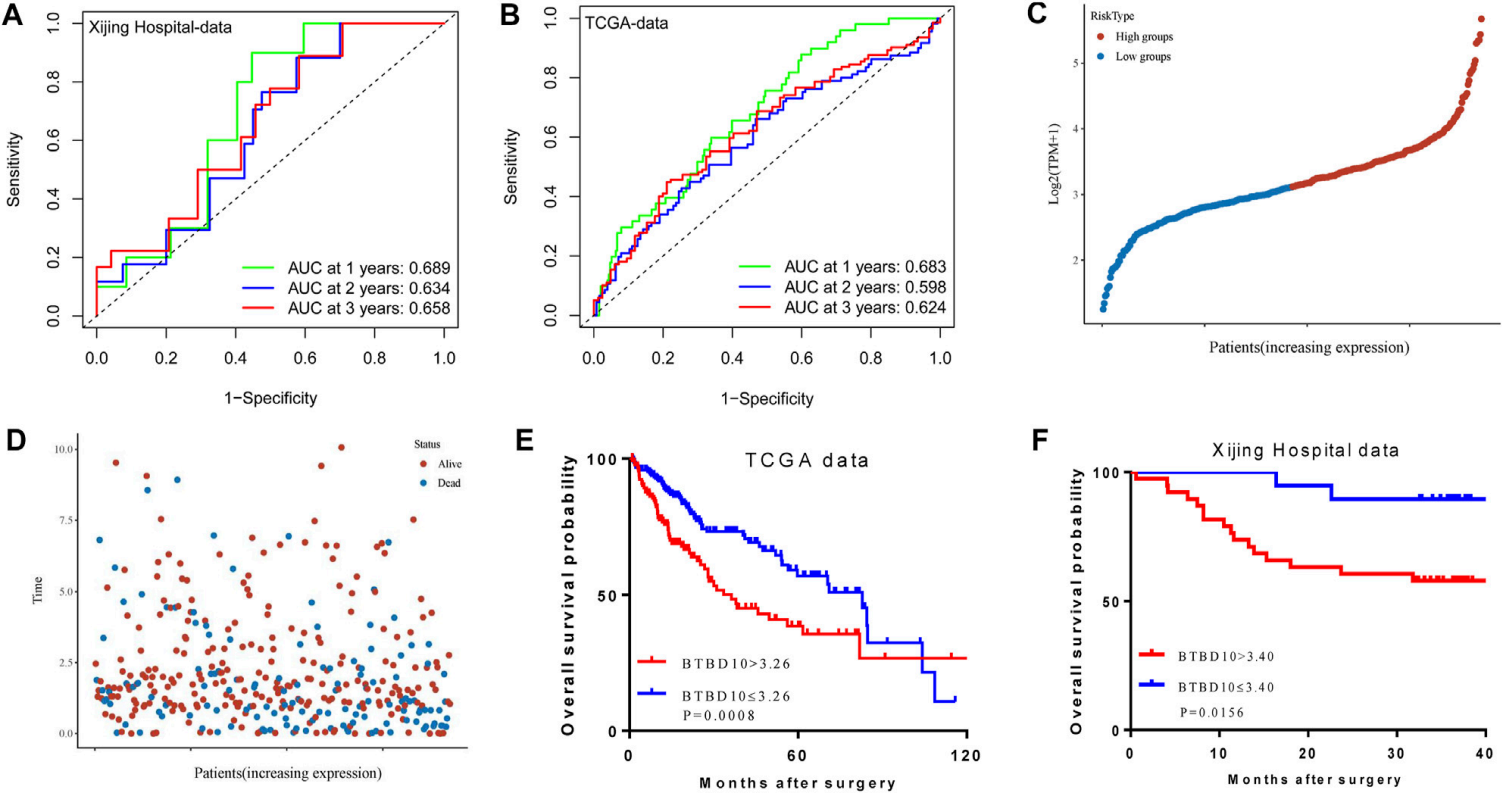
The predictive ability of the BTBD10 for 1, 2, and 3 years: **(A)** ROC curves drawn using TCGA data; **(B)** ROC curves drawn using Xijing Hospital data. In the TCGA data, the expression of BTBD10 was related to the survival time and survival status of patients: **(C)** risk curves; **(D)**survival status distribution map. Kaplan-Meier curve in patients with hepatocellular carcinoma: **(E)** TCGA data; **(F)** Xijing Hospital data.

**FIGURE 4:**
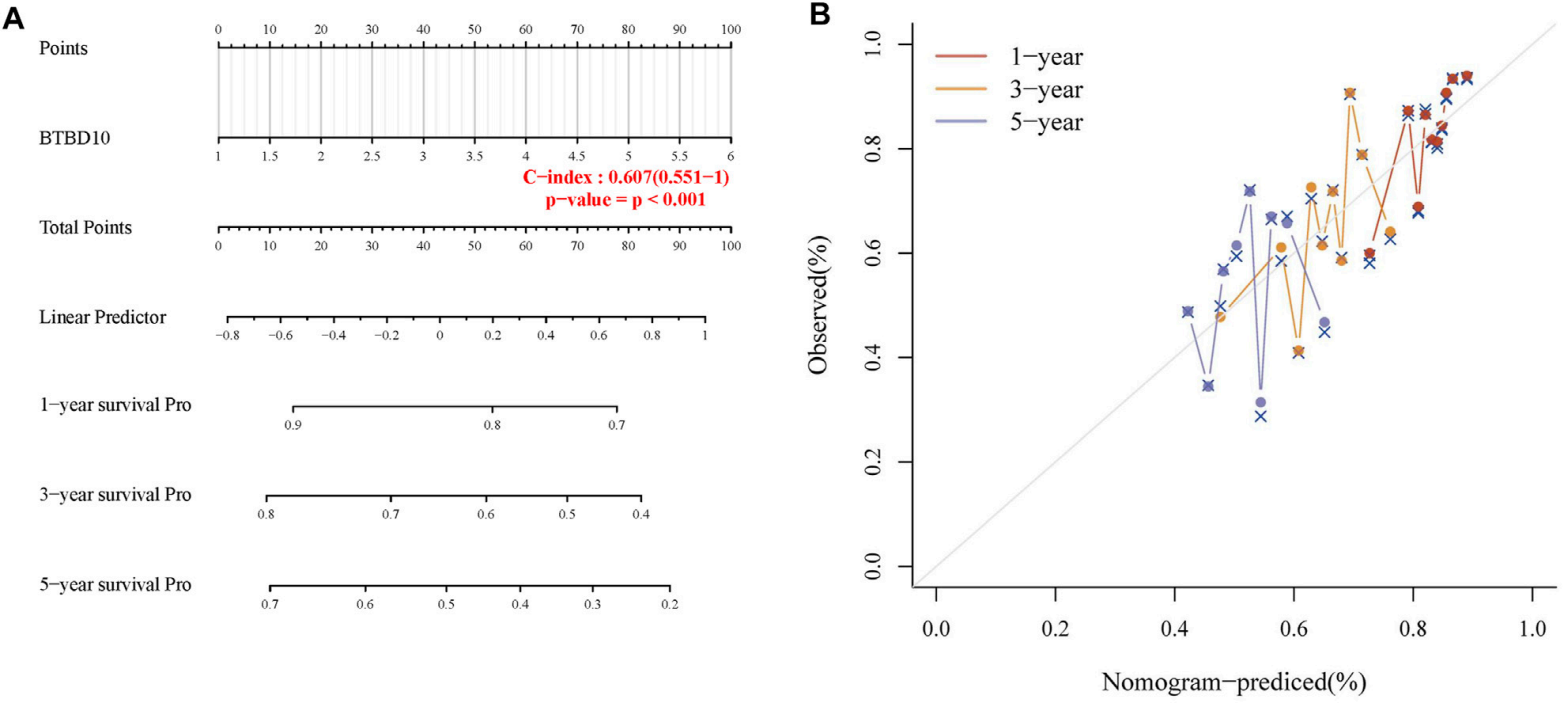
**(A)** Nomogram to predict the 1-year, 3-years and 5-years progression-free survival (PFS) of HCC patients; **(B)** Calibration curve for the PFS nomogram model with dashed diagonal line represents the ideal nomogram.

The area under ROC curve (AUC) of BTBD10 expression based on TCGA data and Xijing Hospital data were 0.689 and 0.683, respectively, suggesting that BTBD10 has a strong predictive ability for the survival of HCC patients (Figures 3A,B). In the distribution of survival status of HCC patients, we observed that with the increase of BTBD10 expression, the number of patients dying increased (Figures 3C,D). K-M curve confirmed that BTBD10 expression was positively correlated with poor prognosis of HCC patients (Figures 3E,F). Univariate and multivariate Cox analysis showed that BTBD10 was an independent prognostic risk factor for HCC patients, with hazard ratios (HR, the high expression divided by the low expression) of 1.750 and 5.088, respectively (Table 2, 3). The expression level of BTBD10, an independent prognostic risk factor, provides a quantitative method for clinicians to predict the likelihood of progression-free survival at 1, 3, and 5 years for HCC patients (Figures 4A,B).

**TABLE 2:** TCGA data: Univariate and multivariate cox regression analyses of risk factors associated with overall survival.

Variables	Univariate analysis		Multivariate analysis	
	HR (95% CI)	P Value	HR (95% CI)	P Value
Sex (male vs. female)	0.830 (0.572–1.203)	0.325	2.767 (1.920–3.986)	**<0.0001**
Age (>70 vs. ≤70)	1.365 (0.909–2.049)	0.133		
Grade (Ⅰ+Ⅱ vs. Ⅲ+Ⅳ)	0.934 (0.638–1.368)	0.726		
TNM (Ⅲ+Ⅳ vs. Ⅰ+Ⅱ)	2.473 (1.709–3.579)	**<0.0001**		
T (Ⅲ+Ⅳ vs. Ⅰ+Ⅱ)	2.850 (1.980–4.104)	**<0.0001**		
BTBD10 (>3.26 vs. ≤3.26)	1.836 (1.281–2.632)	**0.001**	1.750 (1.220–2.512)	**0.002**

Bold value indecates P < 0.05 was statistically significant.

**TABLE 3:** Xijing hospital data: Univariate and multivariate cox regression analyses of risk factors associated with overall survival.

Variables	Univariate analysis		Multivariate analysis	
	HR (95% CI)	P Value	HR (95% CI)	P Value
Sex (male vs. female)	0.592 (0.195–1.798)	0.355	4.875 (1.808–13.144)	**0.002**
Age (>53 vs. ≤53)	0.575 (0.223–1.485)	0.253		
ALT, µ/L (>38 vs.≤38)	0.420 (0.138–1.276)	0.126		
AST, µ/L (>34 vs.≤34)	3.644 (1.297–10.241)	**0.014**		
HBsAg (positive vs negative)	26.717 (0.124–5749.860)	0.231		
TBil, µmol/L, (>14.3 vs.≤14.3)	1.446 (0.543–3.854)	0.461		
AFP, ng/ml (>7.79 vs. ≤7.79)	8.868 (1.179–66.696)	**0.034**		
Liver cirrhosis	0.959 (0.378–2.430)	0.929		
Tumor number (single vs multiple)	0.448 (0.060–3.369)	0.436		
Tumor diameter, cm (>7.1 vs. ≤7. 1)	4.540 (1.752–11.763)	**0.002**		
Tumor capsule	4.302 (1.600–11.563)	**0.004**	5.622 (1.938–16.307)	**0.001**
Cell differentiation (poor/moderate vs. well)	0.974 (0.282–3.367)	0.967		
Microvascular invasion	1.866 (0.665–5.238)	0.236		
TNM (Ⅲ+Ⅳ vs. Ⅰ+Ⅱ)	2.406 (0.857–6.753)	0.095		
BCLC (0 + A VS B + C)	1.523 (0.572–4.060)	0.400		
BTBD10 (>3.40 vs. ≤3.40)	5.095 (1.170–22.187)	**0.003**	5.088 (1.146–22.590)	**0.032**

Bold value indecates P < 0.05 was statistically significant.

**FIGURE 5:**
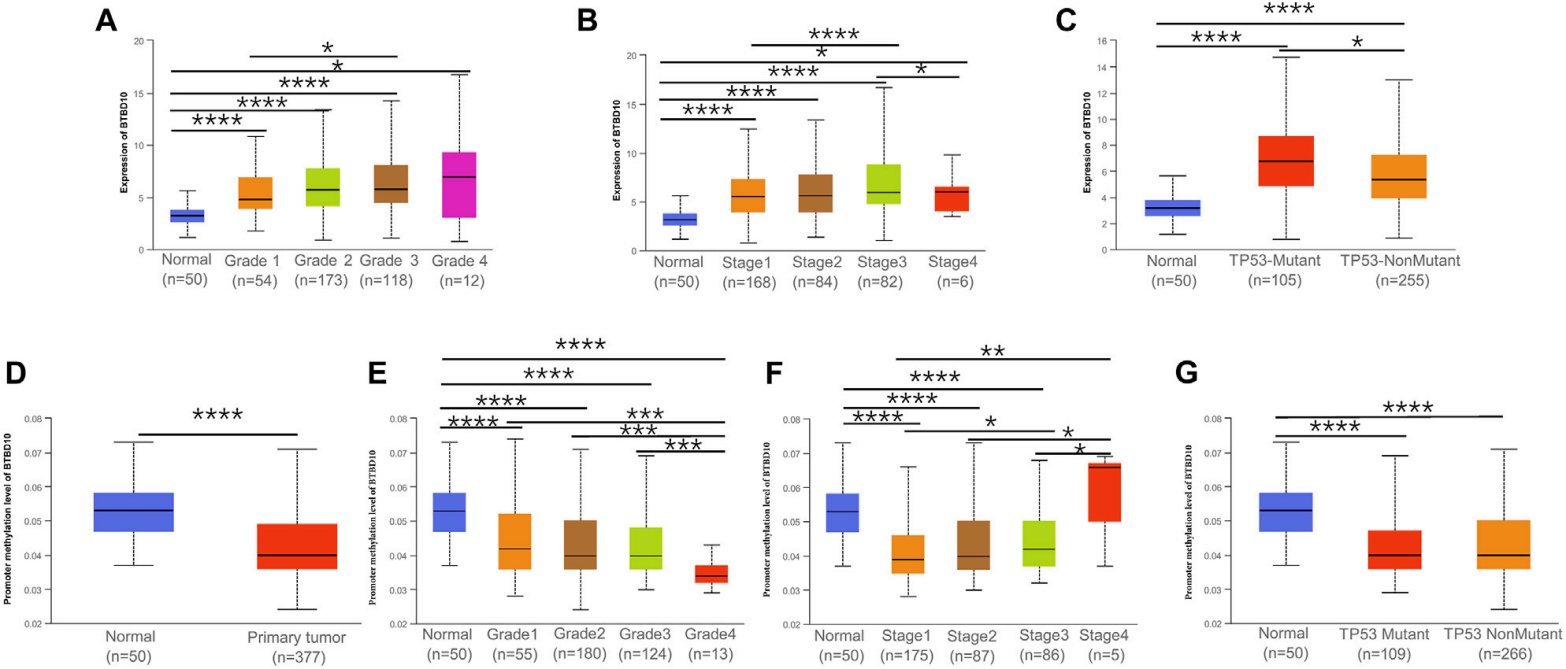
Association between expression of BTBD10 mRNA and clinical characteristics in HCC from the UALCAN database. **(A)** tumor grades; **(B)** tumor stages; **(C)** TP53 mutation. **(D)** Promoter methylation level of BTBD10 in HCC. Association between expression of Promoter methylation level of BTBD10 mRNA and clinical characteristics in HCC from the UALCAN database. **(E)** tumor grades; **(F)** tumor stages; **(G)** TP53 mutation.

In addition, we investigated the correlation between BTBD10 expression and TNM stage and/or grade, TP53 mutation and DNA methylation in HCC to elucidate the potential causes of adverse impact of BTBD10 on the prognosis of HCC patients. The expression of BTBD10 in HCC tissues were associated with different TNM stages and grades of HCC patients (Supplementary Figure S1A). The level of BTBD10 was found to be increased along with the increase of clinical stage and pathological grade of HCC patients (Figures 5A,B). Surprisingly, TP53 mutant group exhibited higher expression of BTBD10 than that of non-mutant group (Figure 5C). Previous studies have shown that gene bodies hypermethylation and gene promoter hypomethylation can lead to the occurrence of tumors, and gene promoter methylation level is negatively correlated with gene expression (Keller et al., 2016; Sun et al., 2018). Our results showed that the expression of BTBD10 promoter methylation in tumor tissues was lower than that in normal tissues, and the level of BTBD10 promoter methylation gradually decreased with the increase of tumor grade **(** Figures 5D,E **)**. However, the methylation level of BTBD10 promoter did not change with tumor stage and TP53 mutation (Figures 5F,G). These results reversely proved that high expression of BTBD10 can promote the progression of HCC.

### Enrichment Analysis

Exploration of the mechanism by which BTBD10 affects HCC progression will help guide clinical treatment. GO and KEGG analysis of TCGA data showed that BTBD10 affected the progression of HCC by regulating cell cycle, transcription, translation and immune response, and the BTBD10 high expression group mainly regulated immune-related pathways (Supplementary Figure S1B).

### Relationship Between TILs and BTBD10

**FIGURE 6:**
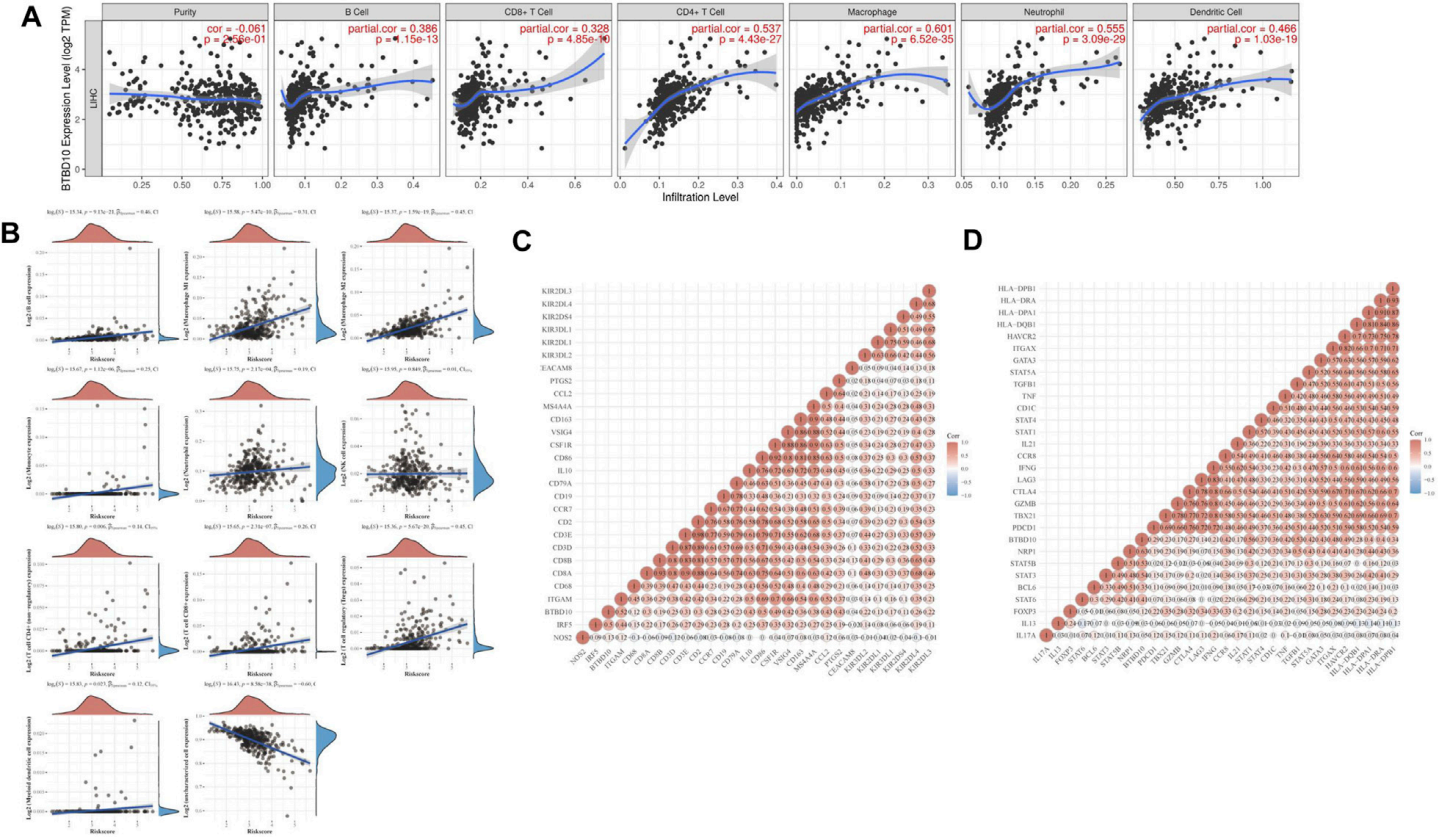
Correlation analysis between BTBD10 expression and levels of infiltrating immune cells in HCC. **(A)** From the TIMER website; **(B)** TCGA data from ACLBI website; **(C,D)** A Heatmap of the correlation between BTBD10 and surface molecules of tumor-infiltrating lymphocytes (in the diagram, red represents positive correlation, blue represents negative correlation).

TIMER and TCGA analysis revealed that BTBD10 expression was positively correlated with intratumoral B cells, CD4^+^ T cells, CD8^+^ T cells, macrophages, neutrophils and dendritic cells (DCs) (Figures 6A,B). In order to provide a more accurate target for immunotherapy of HCC, the correlation between BTBD10 and TIL surface markers was further studied (Figures 6C,D). Statistical analysis results are shown in **additional file1**.

### Immunotherapy

**FIGURE 7:**
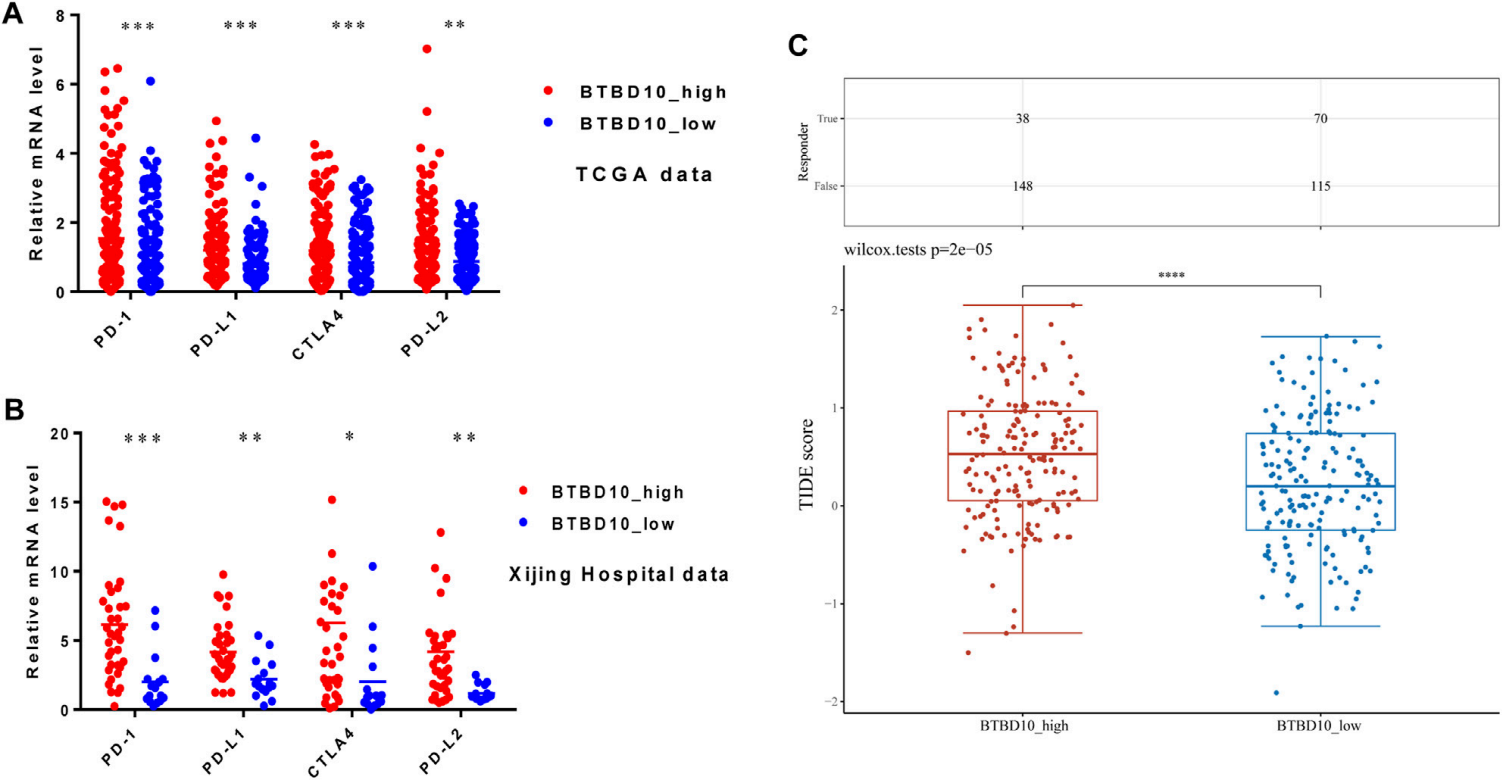
Expression distribution of immune checkpoint genes in different BTBD10 expression groups **(A)**TCGA data; **(B)**Xijing Hospital data; **(C)** TIDE algorithm was used to obtain the statistical table of immune response and the distribution of immune response scores in different groups.

Immunotherapy has gradually replaced surgical resection in the treatment of advanced HCC patients. In the TCGA and Xijing Hospital data, expression levels of PD-1, PD-L1, CTLA4 and PD-L2 in the high BTBD10 expression group were significantly higher than those in the low BTBD10 expression group and the normal group (Figures 7A,B). Subsequently, the Tumor Immune Dysfunction and Exclusion (TIDE) algorithm revealed that the response to ICIs was poorer in the high BTBD10 expression group than in the low BTBD10 expression group (high TIDE score, worse response to ICIs, and short survival after ICIs treatment) (Jiang et al., 2018a) (Figure 7C).

### Correlation Analysis of Between BTBD10 and m6A Methylation

**FIGURE 8:**
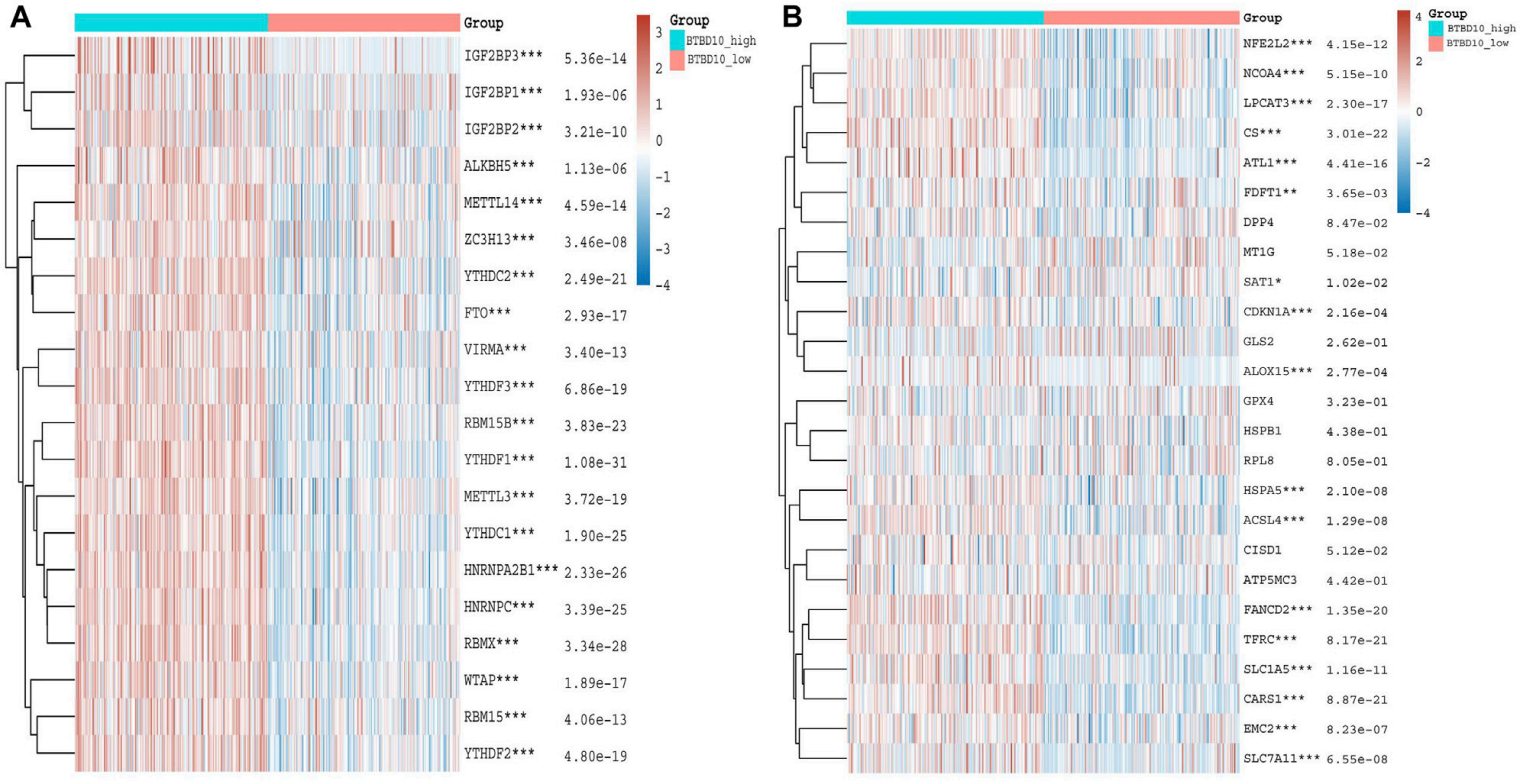
**(A)** Heatmaps of m6A methylation-related genes expression in different BTBD10 expression groups using TCGA data **(B)** Heatmaps of ferroptosis-related genes expression in different BTBD10 expression groups using TCGA data.

In recent years, m6A methylation has become increasingly prominent in anti-tumor immunotherapy. We found that BTBD10 was positively proportional to m6A methylation related genes, including methylation transferases (METTL3, METTL14 and WTAP, etc.), Demethylase (FTO and ALKBH5) and methylated reading proteins (YTHDC1, YThDF1-YTHDF3, IGF2BP1-IGF2BP3, etc.) (Figure 8A). This correlation provides a new perspective for improving the response rate of targeted drugs.

### Correlation Analysis of Between BTBD10 and Ferroptosis

Increased ferroptosis in tumor cells can accelerate cell death and increase the efficacy of immunotherapy. Our results demonstrate that BTBD10 is positively correlated with NFE2L2, NCOA4, LPCAT3, CS, ATL1, FDFT1, CDKN1A, ALOX15, HSPA5, ACSL4, FANCD2, TFRC, SLC1A5, CARS1, EMC2 and SLC7A11, while SAT1 was negatively correlated with BTBD10 (Figure 8B). Patients with high expression of CARS1, FANCD2, SLC1A5, SLC7A11 and TFRC had worse prognosis than those with low expression, prognosis of patients in the SAT1 high expression group was better than that in the low expression group (Supplementary Figure S2). Therefore, low BTBD10 expression group is more suitable for immunization combined with ferroptosis-related treatment.

### IC50 Score

**FIGURE 9:**
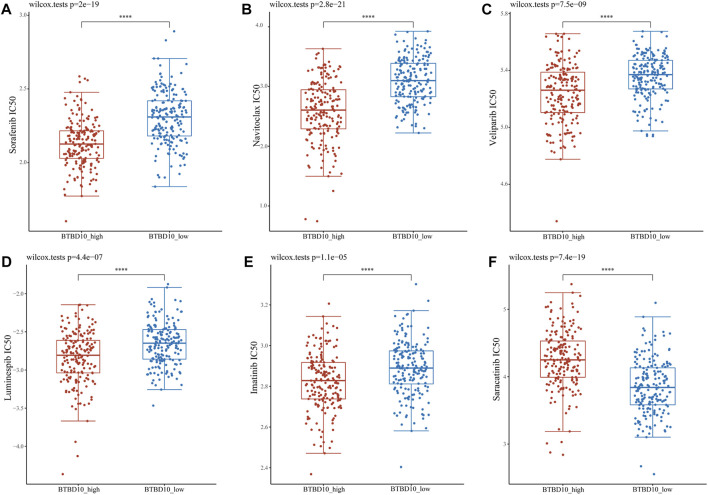
Distribution of IC50 scores of targeted drugs in different BTBD10 expression groups *via* ACLBI website. **(A)** Sorafenib **(B)** Navitoclax **(C)** Veliparib **(D)** Luminespib **(E)** Imatinib **(F)** Saracatinib.

IC50 is an important indicator to evaluate patients’ response to targeted drug therapy. We used GDSC data to predict differences in IC50 scores of chemotherapy agents between BTBD10 expression groups. The IC50 of Sorafenib, Navitoclax, Veliparib, Luminespib and Imatinib was lower in the BTBD10 high-expression group, while the IC50 of Saracatinib was higher (Figure 9). Therefore, these results confirm that the distribution of IC50 of targeted agents in different BTBD10 expression groups is statistically significant.

## Discussion

The KCTD family includes tetramerization (T1) domain containing proteins with diverse biological effects. BTBD10 is a novel member of the KCTD family, which has been found to be an Akt activator, activating Akt by decreasing the protein phosphatase 2A-mediated dephosphorylation (Nawa and Matsuoka, 2013). Studies suggested that BTBD10 inhibited apoptosis of neuronal and islet beta cells *via* the Akt pathway (Wang et al., 2013). The diseases associated with BTBD10 also includes progressive myoclonus epilepsy 1A and progressive myoclonus epilepsy 3. An important paralog of this gene is KCTD20 (Nawa and Matsuoka, 2013). Recently, scientists found that KCTD20) enhanced the proliferation and invasion of NSCLC *via* increasing the phosphorylation level of Akt (Zhang et al., 2017b), indicating a potential role of BTBD10 in tumor diseases. Our study unraveled that BTBD10 was high expressed in HCC tumor and showed an adverse impact on the prognosis of patients with HCC. we confirmed that BTBD10 was associated with tumor clinical stage and pathological grade and can serve as an independent prognostic risk factor for HCC patients.

As TILs in tumor microenvironment (TME) is increasingly regarded as an effective tumor immunotherapy target, revealing its mechanism of action is the premise of individualized precision therapy at present (Wan et al., 2021). Our data suggested that high expression of BTBD10 significantly affected immune response-related pathways, which could reveal the mechanism by which tumor cells evade immune response (Sturm et al., 2019). Analyses of data obtained from TIMER and TCGA data showed that BTBD10 was positively correlated with B cells, CD4^+^ T cells, CD8^+^ T cells, macrophages, DCs and neutrophils in the tumor. BTBD10 interferes with TME homeostasis by affecting the composition ratio of TILs, and this “two-way communication” information exchange promotes tumor progression and metastasis (Postow et al., 2018). TAMs play a mainstay role in the TME and increase tumor invasiveness, which is mainly related to the polarization of M1 and M2 macrophages (Wu et al., 2021). Interferon-γ and other factors trigger the production of inflammatory and immune-stimulating factors by stimulating the polarization of M1 macrophages (Wu et al., 2021). M1 macrophage secretion promotes the epithelial-mesenchymal transition (EMT) of HCC *via* the IL-35 activation of STAT3 (He et al., 2021). IL-35 can also stimulate the polarization of M2 macrophages to induce a mesenchymal-epithelial transition (MET) in HCC cells (He et al., 2021). However, IL-1β secreted by M1 macrophages can increase the expression of PD-L1 in HCC cells, promote tumor progression, and affect the prognosis of tumor patients (Zong et al., 2019). Increasing evidence shows that inflammation is necessary for the initiation and progression of HCC, and when neutrophils are elevated in the TME, HCC is more aggressive and patients have a worse prognosis (Gong et al., 2016). This finding may be related to neutrophil activation of Toll-like receptor 4/9 signaling to increase HCC metastasis *in vivo* (Yang et al., 2020). Furthermore, the C-X-C motif chemokine ligand (CXCL)-2, CXCL8 and CCL25 released by HCC cells can in turn increase the neutrophil ratio in the TME, forming an immunosuppressive microenvironment and leading to tumor progression (Zhu et al., 2020; Xu et al., 2021). In addition, our prediction concluded that BTBD10 may significantly affect the expression of Tregs (FOXP3, CCR8, STAT5B, TGFβ) and T cell exhaustion (PD-1, CTLA4, LAG3, TIM-3) in HCC. These results suggest that BTBD10 may inhibit the recognition of tumor cells by the immune system and help the immune escape of HCC cells. In conclusion, BTBD10 can cause an imbalance in the TME by regulating the ratio of TILs and increase the expression of PD-1/PD-L1 to cause the metastasis and invasion of HCC.

Although many prognostic markers for HCC have been identified, treatment for HCC is not ideal because drug response is usually a complex feature, often influenced by many genomic and environmental factors (Lu et al., 2019). Our results showed that the expression levels of PD-1 and PD-L1 in the high expression group of BTBD10 were significantly higher than those in the low expression group, and patients in the high expression group of BTBD10 had better ORR of targeted drugs than those in the low expression group. However, TIDE score of patients with high expression of BTBD10 was higher than that of patients with low expression of BTBD10 (the higher the TIDE score, the worse the efficacy of ICIs) (Jiang et al., 2018b), which may be because the increased binding between PD-1 and PD-L1 on T cells in TME reduces the immune capacity of T cells and accelerates the depletion of T cells (Abd El Aziz et al., 2020). PD-L1 and CTLA-4 are highly expressed in DCs and B cells in HCC and downregulate the T-cell-mediated immune response through the function of immunosuppression (Zhou et al., 2017). In addition, high PD-L1 expression can increase AFP and TIL levels in patients, providing a new perspective for the regulation of the PD-L1/PD-1 pathway in HCC immunotherapy (Liu et al., 2019). Although targeted drugs have been the treatment standard for patients with advanced HCC, greatly improving the tumor resection rate and survival rate of patients with cancer, but the effective rate of single drugs is only 12–20%, thus limiting their clinical application (Kudo et al., 2018; Abd El Aziz et al., 2020; Pinter et al., 2021).

Therefore, it is urgent to predict and evaluate the efficacy of immunotherapy more accurately in clinical practice to improve the efficacy of precise individualized therapy. Studies have shown that dual immune checkpoint blockade can enhance anti-tumor immunity to HCC(Du et al., 2021). For example, Imatinib reversed Sorafenib induced autophagy, and the combination of Imatinib and Sorafenib had a synergistic effect on HCC cells compared to monotherapy (Xiao et al., 2021). Meanwhile, m6A methyltransferase METTL3 also enhanced Sorafenib’s ability to inhibit HCC by decreasing the autophagy pathway (Lin et al., 2020). However, the demethylase FTO can reduce the expression of METTL3 by demethylating the m6A modified base, and the up-regulation of methyltransferase METTL4 and down-regulation of demethylase FTO can increase the ferroptosis of cells (Shen et al., 2021). Ferroptosis refers to the metabolic disorder of lipid oxides in cells, producing toxic lipids that induce cell death, but this mode of death can be blocked by iron-chelating agents, presenting iron dependence (Li et al., 2021). Sorafenib, as a promoter of ferroptosis, can cause the accumulation of ROS by inhibiting system XC-, leading to the depletion of intracellular glutathione (GSH), thus causing ferroptosis and increasing the response rate of patients to sorafenib (Li et al., 2021). Previous studies have shown that SAT1 participates in the regulation of p53-mediated ROS response and ferroptosis, and enhances the role of p53 in promoting tumor cell apoptosis (Ou et al., 2016). Our study showed that the expression of SAT1 in the high expression group of BTBD10 was lower than that in the low expression group, and TIDE results suggested that the high expression group had a lower ORR to ICIs. Therefore, patients with low BTBD10 expression are more suitable for targeted drugs combined with ferroptosis-related therapy, while patients with high BTBD10 expression are more suitable for multiple targeted drugs combination or combined m6A-related therapy, which may bring hope for the treatment of HCC patients. In summary, BTBD10 showed an advantage over other prognostic markers. However, there are still many unknown factors that limit the clinical application of ICIs, so we need to collect more data in the future to screen out more sensitive ICIs and increase the survival time of HCC patients.

## Conclusion

We demonstrate that BTBD10 is associated with TILs and immune checkpoints, and we illustrate the molecular mechanism by which BTBD10 acts as an independent risk factor in disease progression in HCC patients. Our results suggest that BTBD10 may serve as a prognostic biomarker for HCC.

## Data Availability

The original contributions presented in the study are included in the article/Supplementary Material, further inquiries can be directed to the corresponding authors.

## Supplementary Material

Supplementary Figure S1 (A) Sankey diagram of correlation between BTBD10 and tumor stages, grades and survival status distribution of HCC patients. Each column represents a characteristic variable, different colors represent different types or stages, and lines represent the distribution of the same sample in different characteristic variables. (B) TCGA data were used to predict the signaling pathways that BTBD10 might affect the progression of HCC, and the representative hallmark term GSEA plot was drawn.

Supplementary Figure S2 In TCGA data, effect of ferroptosis-related genes on overall survival in patients with HCC: **(A)** CARS1, **(B)** FANCD2, **(C)** SLC1A5, **(D)** SLC7A11, **(E)** TFRC, and **(F)** SAT1.

## References

[B1] Abd El Aziz M. A. Facciorusso A. Nayfeh T. Saadi S. Elnaggar M. Cotsoglou C. (2020). Immune Checkpoint Inhibitors for Unresectable Hepatocellular Carcinoma. Vaccines (Basel) 8 (4), 8040616. 10.3390/vaccines8040616 PMC7712941 33086471

[B2] Chen J. Xu J. Ying K. Cao G. Hu G. Wang L. (2004). Molecular Cloning and Characterization of a Novel Human BTB Domain-Containing Gene, BTBD10, Which Is Down-Regulated in Glioma. Gene 340 (1), 61–69. 10.1016/j.gene.2004.05.028 15556295

[B3] Chen W. Zheng R. Baade P. D. Zhang S. Zeng H. Bray F. (2016). Cancer Statistics in China, 2015. CA: A Cancer J. Clinicians 66 (2), 115–132. 10.3322/caac.21338 26808342

[B4] Cheon J. Yoo C. Hong J. Y. Kim H. S. Lee D. W. Lee M. A. (2021). Efficacy and Safety of Atezolizumab Plus Bevacizumab in Korean Patients with Advanced Hepatocellular Carcinoma[J]. Liver Int. 10.1111/liv.15102 34792284

[B5] D'Avola D. Granito A. de la Torre-Alaez M. Piscaglia F. (2021). The Importance of Liver Functional reserve in the Non-surgical Treatment of Hepatocellular Carcinoma[J]. J. Hepatol. 10.1016/j.jhep.2021.11.013 34793869

[B6] Du Y. Zhang D. Wang Y. Wu M. Zhang C. Zheng Y. (2021). A Highly Stable Multifunctional Aptamer for Enhancing Antitumor Immunity against Hepatocellular Carcinoma by Blocking Dual Immune Checkpoints. Biomater. Sci. 9 (11), 4159–4168. 10.1039/d0bm02210a 33970170

[B7] Gong Z.-J. Guo W. Sun Y.-F. Zhang X. Qiu S.-J. Zhou J. (2016). Prognostic Value of Fever Grade Combined with Neutrophil Percentage in Hepatocellular Carcinoma Patients Presenting Fever as the Initial Manifestation. Onco Targets Ther 9, 6281–6290. 10.2147/ott.s109023 27789960 PMC5072517

[B8] Gunasekaran G. Bekki Y. Lourdusamy V. Schwartz M. (2021). Surgical Treatments of Hepatobiliary Cancers. Hepatology 73 (Suppl. 1), 128–136. 10.1002/hep.31325 32438491

[B9] He Y. Pei J.-h. Li X.-q. Chi G. (2021). IL-35 Promotes EMT through STAT3 Activation and Induces MET by Promoting M2 Macrophage Polarization in HCC. Biochem. biophysical Res. Commun. 559, 35–41. 10.1016/j.bbrc.2021.04.050 33932898

[B10] Hughes C. B. Humar A. (2021). Liver Transplantation: Current and Future. Abdom. Radiol. 46 (1), 2–8. 10.1007/s00261-019-02357-w 31953588

[B11] Jiang P. Gu S. Pan D. Fu J. Sahu A. Hu X. (2018). Signatures of T Cell Dysfunction and Exclusion Predict Cancer Immunotherapy Response. Nat. Med. 24 (10), 1550–1558. 10.1038/s41591-018-0136-1 30127393 PMC6487502

[B12] Jiang P. Gu S. Pan D. Fu J. Sahu A. Hu X. (2018). Signatures of T Cell Dysfunction and Exclusion Predict Cancer Immunotherapy Response. Nat. Med. 24 (10), 1550–1558. 10.1038/s41591-018-0136-1 30127393 PMC6487502

[B13] Keller T. E. Han P. Yi S. V. (2016). Evolutionary Transition of Promoter and Gene Body DNA Methylation across Invertebrate-Vertebrate Boundary. Mol. Biol. Evol. 33 (4), 1019–1028. 10.1093/molbev/msv345 26715626 PMC4776710

[B14] Kudo M. Finn R. S. Qin S. Han K.-H. Ikeda K. Piscaglia F. (2018). Lenvatinib versus Sorafenib in First-Line Treatment of Patients with Unresectable Hepatocellular Carcinoma: a Randomised Phase 3 Non-inferiority Trial. The Lancet 391 (10126), 1163–1173. 10.1016/s0140-6736(18)30207-1 29433850

[B15] Li Y. Xia J. Shao F. Zhou Y. Yu J. Wu H. (2021). Sorafenib Induces Mitochondrial Dysfunction and Exhibits Synergistic Effect with Cysteine Depletion by Promoting HCC Cells Ferroptosis. Biochem. biophysical Res. Commun. 534, 877–884. 10.1016/j.bbrc.2020.10.083 33162029

[B16] Lin Z. Niu Y. Wan A. Chen D. Liang H. Chen X. (2020). RNA M6 A Methylation Regulates Sorafenib Resistance in Liver Cancer through FOXO3-Mediated Autophagy. EMBO J. 39 (12), e103181. 10.15252/embj.2019103181 32368828 PMC7298296

[B17] Liu G.-M. Li X.-G. Zhang Y.-M. (2019). Prognostic Role of PD-L1 for HCC Patients after Potentially Curative Resection: a Meta-Analysis. Cancer Cel Int 19, 22. 10.1186/s12935-019-0738-9 PMC6352338 30718977

[B18] Lu X. Jiang L. Zhang L. Zhu Y. Hu W. Wang J. (2019). Immune Signature-Based Subtypes of Cervical Squamous Cell Carcinoma Tightly Associated with Human Papillomavirus Type 16 Expression, Molecular Features, and Clinical Outcome. Neoplasia 21 (6), 591–601. 10.1016/j.neo.2019.04.003 31055200 PMC6658934

[B19] Nawa M. Kage-Nakadai E. Aiso S. Okamoto K. Mitani S. Matsuoka M. (2012). Reduced Expression of BTBD10, an Akt Activator, Leads to Motor Neuron Death. Cell Death Differ 19 (8), 1398–1407. 10.1038/cdd.2012.19 22388351 PMC3392628

[B20] Nawa M. Kanekura K. Hashimoto Y. Aiso S. Matsuoka M. (2008). A Novel Akt/PKB-Interacting Protein Promotes Cell Adhesion and Inhibits Familial Amyotrophic Lateral Sclerosis-Linked Mutant SOD1-Induced Neuronal Death via Inhibition of PP2A-Mediated Dephosphorylation of Akt/PKB. Cell Signal. 20 (3), 493–505. 10.1016/j.cellsig.2007.11.004 18160256

[B21] Nawa M. Matsuoka M. (2013). KCTD20, a Relative of BTBD10, Is a Positive Regulator of Akt. BMC Biochem. 14, 27. 10.1186/1471-2091-14-27 24156551 PMC3827329

[B22] Ou Y. Wang S.-J. Li D. Chu B. Gu W. (2016). Activation of SAT1 Engages Polyamine Metabolism with P53-Mediated Ferroptotic Responses. Proc. Natl. Acad. Sci. USA 113 (44), E6806–E6812. 10.1073/pnas.1607152113 27698118 PMC5098629

[B23] Pérez-Romasanta L. A. González-Del Portillo E. Rodríguez-Gutiérrez A. Matías-Pérez Á. (2021). Stereotactic Radiotherapy for Hepatocellular Carcinoma, Radiosensitization Strategies and Radiation-Immunotherapy Combination. Cancers (Basel) 13 (2), 192. 10.3390/cancers13020192 PMC7825787 33430362

[B24] Pinter M. Scheiner B. Peck-Radosavljevic M. (2021). Immunotherapy for Advanced Hepatocellular Carcinoma: a Focus on Special Subgroups. Gut 70 (1), 204–214. 10.1136/gutjnl-2020-321702 32747413 PMC7788203

[B25] Postow M. A. Sidlow R. Hellmann M. D. (2018). Immune-Related Adverse Events Associated with Immune Checkpoint Blockade. N. Engl. J. Med. 378 (2), 158–168. 10.1056/nejmra1703481 29320654

[B26] Shen M. Li Y. Wang Y. Shao J. Zhang F. Yin G. (2021). N6-methyladenosine Modification Regulates Ferroptosis through Autophagy Signaling Pathway in Hepatic Stellate Cells. Redox Biol. 47, 102151. 10.1016/j.redox.2021.102151 34607160 PMC8495178

[B27] Sturm G. Finotello F. Petitprez F. Zhang J. D. Baumbach J. Fridman W. H. (2019). Comprehensive Evaluation of Transcriptome-Based Cell-type Quantification Methods for Immuno-Oncology. Bioinformatics (Oxford, England) 35 (14), i436–i445. 10.1093/bioinformatics/btz363 PMC6612828 31510660

[B28] Sun W. Bunn P. Jin C. Little P. Zhabotynsky V. Perou C. M. (2018). The Association between Copy Number Aberration, DNA Methylation and Gene Expression in Tumor Samples. Nucleic Acids Res. 46 (6), 3009–3018. 10.1093/nar/gky131 29529299 PMC5887505

[B29] Sung H. Ferlay J. Siegel R. L. Laversanne M. Soerjomataram I. Jemal A. (2021). Global Cancer Statistics 2020: GLOBOCAN Estimates of Incidence and Mortality Worldwide for 36 Cancers in 185 Countries. CA A. Cancer J. Clin. 71 (3), 209–249. 10.3322/caac.21660 33538338

[B30] Wan P. K.-T. Ryan A. J. Seymour L. W. (2021). Beyond Cancer Cells: Targeting the Tumor Microenvironment with Gene Therapy and Armed Oncolytic Virus. Mol. Ther. 29 (5), 1668–1682. 10.1016/j.ymthe.2021.04.015 33845199 PMC8116634

[B31] Wang X. Gong Y. Zheng M. Xie Q. Tang H. Wang D. (2013). Early Changes in GMRP1 after Intracerebral Hemorrhage: Involvement in Brain Damage and Cell Apoptosis. Acta Neurochir Suppl. 118, 163–167. 10.1007/978-3-7091-1434-6_30 23564125

[B32] Wang X. Liu Y. Yang Z. Zhang Z. Zhou W. Ye Z. (2011). Glucose Metabolism-Related Protein 1 (GMRP1) Regulates Pancreatic Beta Cell Proliferation and Apoptosis via Activation of Akt Signalling Pathway in Rats and Mice. Diabetologia 54 (4), 852–863. 10.1007/s00125-011-2048-1 21267538

[B33] Wu J. Gao W. Tang Q. Yu Y. You W. Wu Z. (2021). Retracted: M2 Macrophage-Derived Exosomes Facilitate HCC Metastasis by Transferring α M β 2 Integrin to Tumor Cells. Hepatology 73 (4), 1365–1380. 10.1002/hep.31432 32594528 PMC8360085

[B34] Xiao M.-C. Qian H. Huang C.-K. Zheng B.-N. Yan F.-Z. Liu F. (2021). Imatinib Inhibits the Malignancy of Hepatocellular Carcinoma by Suppressing Autophagy. Eur. J. Pharmacol. 906, 174217. 10.1016/j.ejphar.2021.174217 34087223

[B35] Xu X. Ye L. Zhang Q. Shen H. Li S. Zhang X. (2021). Group-2 Innate Lymphoid Cells Promote Hepatocellular Carcinoma Progression via CXCL2-Neutrophil Induced Immunosuppression[J]. Hepatology 74 (5), 2526–2543. 33829508 10.1002/hep.31855 PMC8597094

[B36] Yang L.-Y. Luo Q. Lu L. Zhu W.-W. Sun H.-T. Wei R. (2020). Increased Neutrophil Extracellular Traps Promote Metastasis Potential of Hepatocellular Carcinoma via Provoking Tumorous Inflammatory Response. J. Hematol. Oncol. 13 (1), 3. 10.1186/s13045-019-0836-0 31907001 PMC6945602

[B37] Zhang X. Zhou H. Cai L. Fan C. Liu Y. Wang L. (2017). Kctd20 Promotes the Development of Non-small Cell Lung Cancer through Activating Fak/AKT Pathway and Predicts Poor Overall Survival of Patients. Mol. Carcinog 56 (9), 2058–2065. 10.1002/mc.22660 28398603

[B38] Zhang X. Zhou H. Cai L. Fan C. Liu Y. Wang L. (2017). Kctd20 Promotes the Development of Non-small Cell Lung Cancer through Activating Fak/AKT Pathway and Predicts Poor Overall Survival of Patients. Mol. Carcinog 56 (9), 2058–2065. 10.1002/mc.22660 28398603

[B39] Zhou G. Sprengers D. Boor P. P. C. Doukas M. Schutz H. Mancham S. (2017). Antibodies against Immune Checkpoint Molecules Restore Functions of Tumor-Infiltrating T Cells in Hepatocellular Carcinomas. Gastroenterology 153 (4), 1107–1119. e1110. 10.1053/j.gastro.2017.06.017 28648905

[B40] Zhu Q. Pan Q.-Z. Zhong A.-L. Hu H. Zhao J.-J. Tang Y. (2020). Annexin A3 Upregulates the Infiltrated Neutrophil-Lymphocyte Ratio to Remodel the Immune Microenvironment in Hepatocellular Carcinoma. Int. immunopharmacology 89, 107139. 10.1016/j.intimp.2020.107139 33191179

[B41] Zong Z. Zou J. Mao R. Ma C. Li N. Wang J. (2019). M1 Macrophages Induce PD-L1 Expression in Hepatocellular Carcinoma Cells through IL-1β Signaling. Front. Immunol. 10, 1643. 10.3389/fimmu.2019.01643 31379842 PMC6648893

