# Supplementary material for: BTBD10 is a Prognostic Biomarker Correlated With Immune Infiltration in Hepatocellular Carcinoma
Source: Front Mol Biosci. 2022 Jan 4;8:762541. doi: 10.3389/fmolb.2021.762541 (PMC8764259; doi:10.3389/fmolb.2021.762541)
Supplement: Supplementary file 1 [file Table1.DOCX]

| **Additional file1** Correlation analysis between BTBD10 and relate genes and markers of immune cells | | | | | | | | | | |
| --- | --- | --- | --- | --- | --- | --- | --- | --- | --- | --- |
|  | TIMER database analysis | | | | |  | GEPIA database analysis | | | |
| Description | Gene markers | LIHC | | | |  | LIHC | | | |
|  |  | None | | Purity | |  | Tumor | | Normal | |
|  |  | Cor | P | Cor | P |  | Cor | P | Cor | P |
| CD8+T cell | CD8A | 0.272 | 1.14e−07 | 0.287 | 5.85e−08 |  | 0.25 | 1.2e−06 | 0.61 | 2.8e−06 |
|  | CD8B | 0.162 | 1.76e−03 | 0.171 | 1.47e−03 |  | 0.16 | 0.002 | 0.51 | 0.00016 |
| T cell(general) | CD3D | 0.23 | 8.38e−06 | 0.248 | 3.12e−06 |  | 0.16 | 0.0021 | 0.53 | 6.5e−05 |
|  | CD3E | 0.272 | 1.15e−07 | 0.302 | 9.86e−09 |  | 0.25 | 1e−06 | 0.56 | 2e−05 |
|  | CD2 | 0.255 | 6.88e−07 | 0.284 | 8.09e−08 |  | 0.24 | 4.4e−06 | 0.6 | 5.1e−06 |
| B cell | CD19 | 0.258 | 4.71e−07 | 0.244 | 4.61e−06 |  | 0.24 | 2.6e−06 | 0.44 | 0.0014 |
|  | CD79A | 0.195 | 1.52e−04 | 0.195 | 2.64e−04 |  | 0.19 | 0.00019 | 0.58 | 1e−05 |
| Monocyte | CD86 | 0.449 | 0 | 0.507 | 5.69e−24 |  | 0.46 | 1e−20 | 0.65 | 3.9e−07 |
|  | CD115(CSF1R) | 0.406 | 0 | 0.454 | 6.14e−19 |  | 0.46 | 2.9e−20 | 0.59 | 7.5e−06 |
| TAM | CCL2 | 0.375 | 9.48e−14 | 0.405 | 4.74e−15 |  | 0.37 | 3.4e−13 | 0.46 | 0.00076 |
|  | CD68 | 0.366 | 4.36e−13 | 0.377 | 4.03e−13 |  | 0.39 | 3.8e−15 | 0.67 | 9.9e−08 |
|  | IL10 | 0.377 | 5.25e−14 | 0.394 | 2.89e−14 |  | 0.37 | 3.3e−13 | 0.48 | 0.00049 |
| M1 Macrophage | INOS(NOS2) | 0.222 | 1.55e−05 | 0.219 | 4.09e−05 |  | 0.26 | 4.9e−07 | 0.34 | 0.015 |
|  | IRF5 | 0.515 | 1.50e−26 | 0.519 | 3.92e−25 |  | 0.51 | 2.2e−25 | 0.4 | 0.004 |
|  | COX2(PTGS2) | 0.444 | 2.36e−19 | 0.499 | 4.03e−23 |  | 0.49 | 1.2e−23 | 0.54 | 4.4e−05 |
| M2 Macrophage | CD163 | 0.336 | 2.91e−11 | 0.363 | 3.30e−12 |  | 0.16 | 0.0023 | 0.49 | 0.00034 |
|  | VSIG4 | 0.353 | 3.22e−12 | 0.387 | 9.61e−14 |  | 0.35 | 7.4e−12 | 0.51 | 0.00016 |
|  | MS4A4A | 0.331 | 7.69e−11 | 0.372 | 8.52e−13 |  | 0.33 | 6.9e−11 | 0.57 | 1.3e−05 |
| Neutrophils | CD66 b(CEACAM8) | 0.097 | 6.15e−02 | 0.103 | 5.58e−02 |  | 0.1 | 0.05 | 0.48 | 0.00038 |
|  | CD11b(ITGAM) | 0.447 | 0 | 0.475 | 7.46e−21 |  | 0.49 | 3.2e−23 | 0.67 | 8.5e−08 |
|  | CCR7 | 0.274 | 8.08e−08 | 0.295 | 2.37e−08 |  | 0.28 | 2.6e−08 | 0.61 | 2.4e−06 |
| Natural killer cell | KIR2DL1 | 0.033 | 5.20e−01 | 0.009 | 8.65e−01 |  | 0.12 | 0.021 | 0.3 | 0.032 |
|  | KIR2DL3 | 0.223 | 1.40e−05 | 0.221 | 3.37e−05 |  | 0.24 | 2.6e−06 | 0.29 | 0.039 |
|  | KIR2DL4 | 0.191 | 2.10e−04 | 0.196 | 2.57e−04 |  | 0.24 | 2.7e−06 | 0.25 | 0.075 |
|  | KIR3DL1 | 0.107 | 3.95e−02 | 0.116 | 3.09e−02 |  | 0.023 | 0.67 | 0.42 | 0.0027 |
|  | KIR3DL2 | 0.118 | 2.32e−02 | 0.13 | 1.58e−02 |  | 0.27 | 1.5e−07 | 0.08 | 0.58 |
|  | KIR3DL3 | 0.026 | 6.22e−01 | 0.004 | 9.36e−01 |  | 0.12 | 0.017 | 0.35 | 0.014 |
|  | KIR2DS4 | 0.106 | 4.20e−02 | 0.111 | 3.95e−02 |  | 0.095 | 0.068 | 0.35 | 0.012 |
| Dendritic cell | HLA-DPB1 | 0.321 | 3.02e−10 | 0.344 | 5.44e−11 |  | 0.34 | 1.1e−11 | 0.55 | 3.7e−05 |
|  | HLA-DQB1 | 0.216 | 2.91e−05 | 0.231 | 1.40e−05 |  | 0.07 | 0.18 | 0.28 | 0.048 |
|  | HLA-DRA | 0.374 | 1.32e−13 | 0.403 | 6.96e−15 |  | 0.38 | 4.7e−14 | 0.6 | 4.1e−06 |
|  | HLA-DPA1 | 0.369 | 2.98e−13 | 0.403 | 7.12e−15 |  | 0.37 | 9.2e−14 | 0.53 | 7.9e−05 |
|  | BCDA-1(CD1C) | 0.326 | 1.29e−10 | 0.34 | 9.38e−11 |  | 0.32 | 1.7e−10 | 0.4 | 0.0044 |
|  | BDCA-4(NRP1) | 0.598 | 0 | 0.599 | 5.19e−35 |  | 0.57 | 1.4e−33 | 0.73 | 2.5e−09 |
|  | CD11c(ITGAX) | 0.453 | 0 | 0.498 | 5.33e−23 |  | 0.48 | 3.5e−22 | 0.58 | 9.9e−06 |
| Th1 | T-bet(TBX21) | 0.188 | 2.77e−04 | 0.189 | 4.12e−04 |  | 0.19 | 0.00021 | 0.61 | 2.2e−06 |
|  | STAT4 | 0.309 | 1.48e−09 | 0.323 | 8.43e−10 |  | 0.33 | 1.5e−10 | 0.64 | 7.2e−07 |
|  | STAT1 | 0.558 | 0 | 0.565 | 1.76e−30 |  | 0.56 | 2.2e−32 | 0.69 | 3.9e−08 |
|  | IFN-γ(IFNG) | 0.223 | 1.49e−05 | 0.239 | 6.90e−06 |  | 0.21 | 3.7e−05 | 0.52 | 0.00011 |
|  | TNF-α(TNF) | 0.377 | 5.75e−14 | 0.4 | 1.13e−14 |  | 0.39 | 5.4e−15 | 0.48 | 0.00047 |
| Th2 | GATA3 | 0.374 | 8.67e−14 | 0.422 | 2.29e−16 |  | 0.41 | 1.6e−16 | 0.6 | 4.7e−06 |
|  | STAT6 | 0.405 | 4.46e−16 | 0.385 | 1.31e−13 |  | 0.48 | 2.3e−22 | 0.69 | 3.7e−08 |
|  | STAT5A | 0.424 | 1.18e−17 | 0.433 | 3.54e−17 |  | 0.47 | 1e−21 | 0.64 | 4.9e−07 |
|  | IL13 | 0.122 | 1.89e−02 | 0.115 | 3.24e−02 |  | 0.15 | 0.0043 | 0.24 | 0.099 |
| Tfh | BCL6 | 0.374 | 1.25e−13 | 0.366 | 2.33e−12 |  | 0.42 | 8e−17 | 0.38 | 0.0071 |
|  | IL21 | 0.148 | 4.33e−03 | 0.151 | 5.07e−03 |  | 0.11 | 0.03 | 0.21 | 0.14 |
| Th17 | STAT3 | 0.513 | 0 | 0.52 | 2.71e−25 |  | 0.55 | 3.5e−30 | 0.48 | 0.00038 |
|  | IL17A | 0.089 | 8.61e−02 | 0.098 | 6.83e−02 |  | 0.09 | 0.084 | 0.2 | 0.16 |
| Treg | FOXP3 | 0.3 | 3.78e−09 | 0.312 | 3.32e−09 |  | 0.2 | 0.00011 | 0.25 | 0.078 |
|  | CCR8 | 0.551 | 8.33e−31 | 0.584 | 5.43e−33 |  | 0.52 | 7e−27 | 0.48 | 0.00047 |
|  | STAT5B | 0.571 | 0 | 0.586 | 3.82e−33 |  | 0.6 | 8.9e−38 | 0.55 | 3.2e−05 |
|  | TGFβ(TGFB1) | 0.482 | 0 | 0.514 | 1.16e−24 |  | 0.39 | 4.2e−15 | 0.59 | 7.2e−06 |
| T cell exhaustion | PD-1(PDCD1) | 0.287 | 1.91e−08 | 0.286 | 6.69e−08 |  | 0.27 | 9.2e−08 | 0.58 | 8.5e−06 |
|  | CTLA4 | 0.251 | 1.00e−06 | 0.269 | 3.82e−07 |  | 0.25 | 7.5e−07 | 0.42 | 0.0023 |
|  | LAG3 | 0.154 | 3.04e−03 | 0.155 | 4.01e−03 |  | 0.051 | 0.32 | 0.17 | 0.24 |
|  | TIM-3(HAVCR2) | 0.448 | 0 | 0.514 | 1.29e−24 |  | 0.44 | 3.1e−19 | 0.65 | 3.3e−07 |
|  | GZMB | 0.109 | 3.60e−02 | 0.091 | 9.13e−02 |  | 0.037 | 0.48 | 0.43 | 0.0017 |
