# Supplementary figures and images for: BTBD10 is a Prognostic Biomarker Correlated With Immune Infiltration in Hepatocellular Carcinoma
Source: Front Mol Biosci. 2022 Jan 4;8:762541. doi: 10.3389/fmolb.2021.762541 (PMC8764259; doi:10.3389/fmolb.2021.762541)

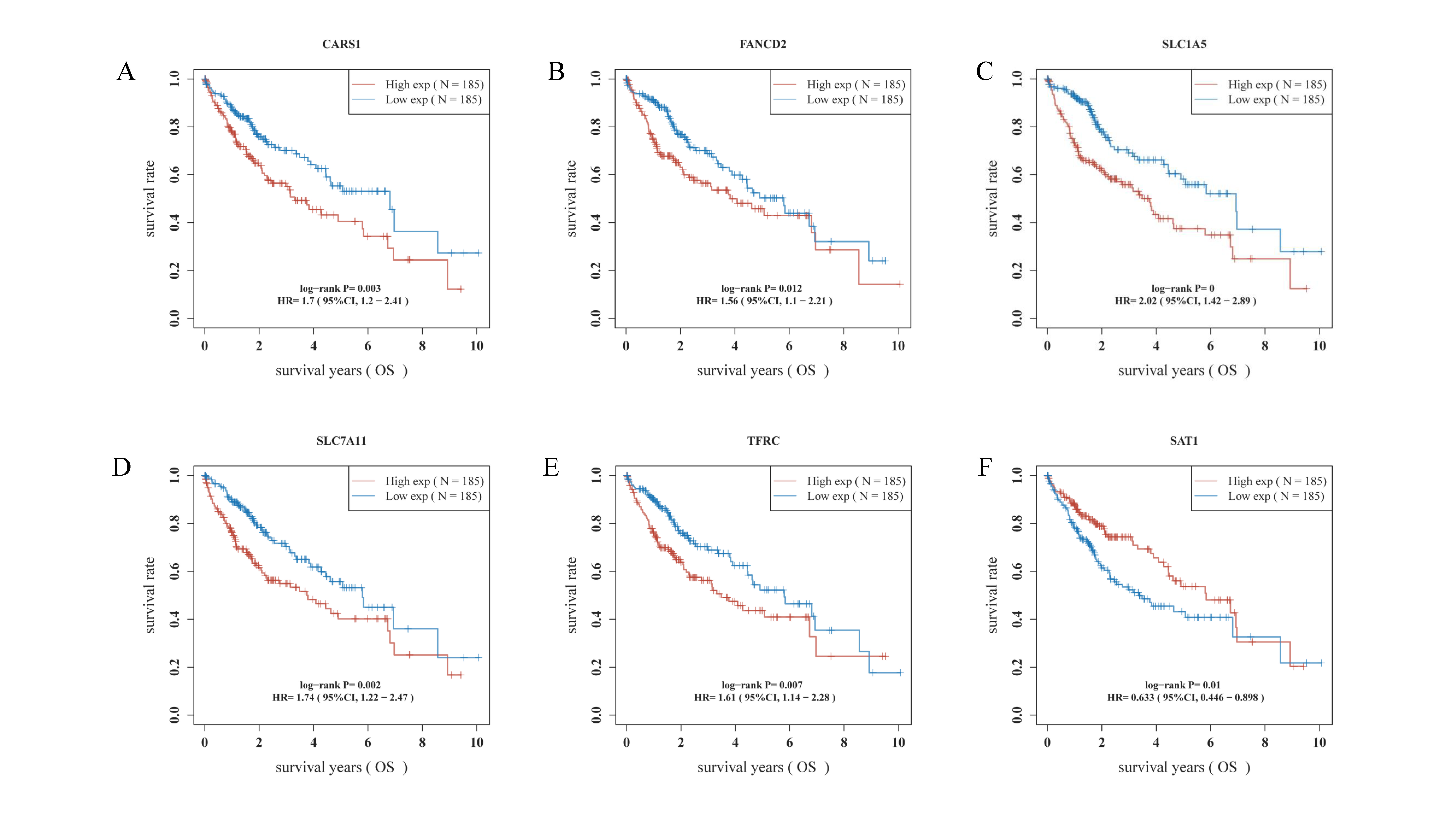

Supplement: Supplementary file 2 [file Image2.tif]

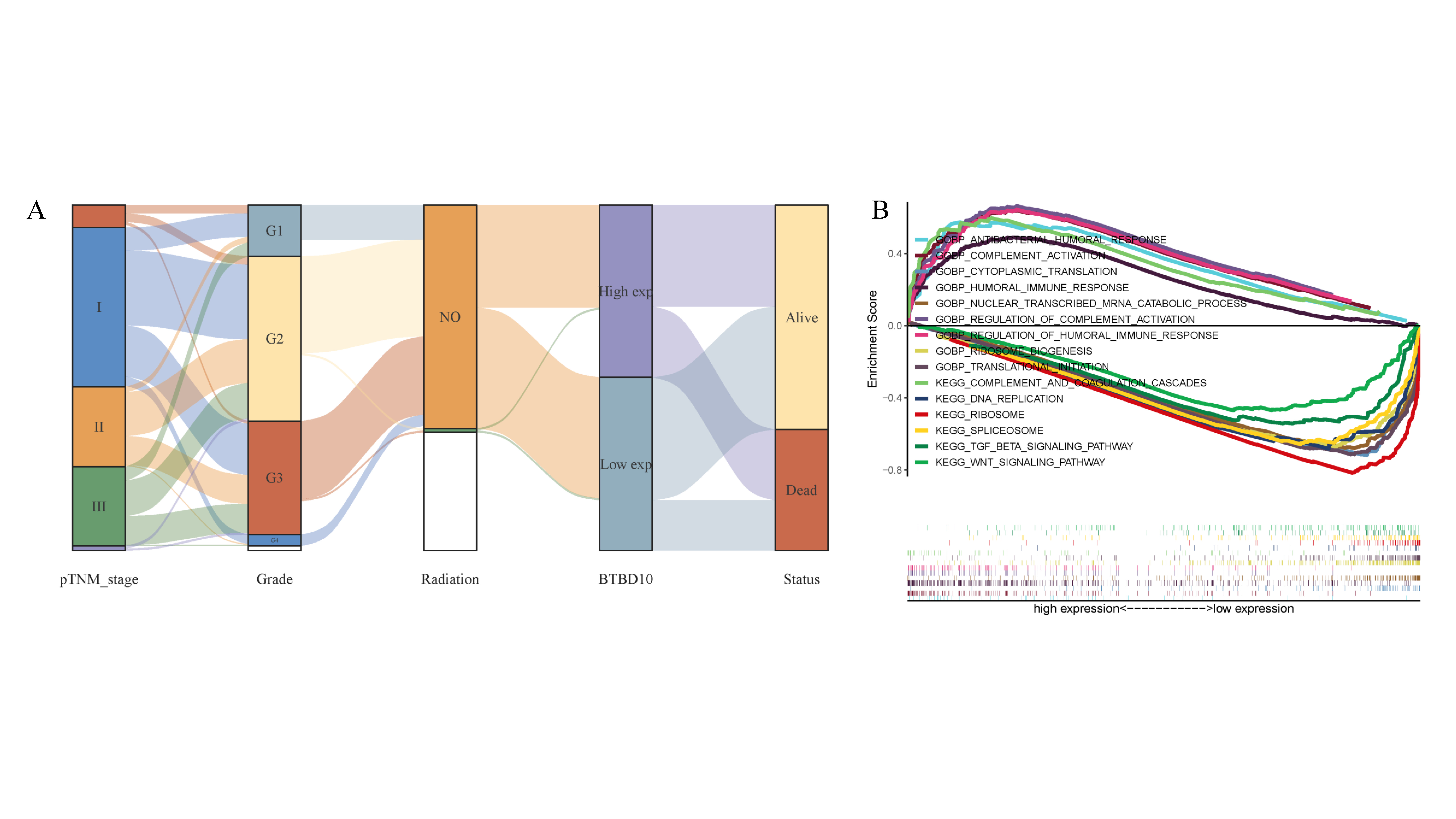

Supplement: Supplementary file 3 [file Image1.tif]
